# FilmArray, an Automated Nested Multiplex PCR System for Multi-Pathogen Detection: Development and Application to Respiratory Tract Infection

**DOI:** 10.1371/journal.pone.0026047

**Published:** 2011-10-19

**Authors:** Mark A. Poritz, Anne J. Blaschke, Carrie L. Byington, Lindsay Allen, Kody Nilsson, David E. Jones, Stephanie A. Thatcher, Thomas Robbins, Beth Lingenfelter, Elizabeth Amiott, Amy Herbener, Judy Daly, Steven F. Dobrowolski, David H. -F. Teng, Kirk M. Ririe

**Affiliations:** 1 Idaho Technology, Inc., Salt Lake City, Utah, United States of America; 2 Department of Pediatrics, University of Utah School of Medicine, Salt Lake City, Utah, United States of America; 3 Primary Children's Medical Center and Department of Pathology, University of Utah, Salt Lake City, Utah, United States of America; University Hospital San Giovanni Battista di Torino, Italy

## Abstract

The ideal clinical diagnostic system should deliver rapid, sensitive, specific and reproducible results while minimizing the requirements for specialized laboratory facilities and skilled technicians. We describe an integrated diagnostic platform, the “FilmArray”, which fully automates the detection and identification of multiple organisms from a single sample in about one hour. An unprocessed biologic/clinical sample is subjected to nucleic acid purification, reverse transcription, a high-order nested multiplex polymerase chain reaction and amplicon melt curve analysis. Biochemical reactions are enclosed in a disposable pouch, minimizing the PCR contamination risk. FilmArray has the potential to detect greater than 100 different nucleic acid targets at one time. These features make the system well-suited for molecular detection of infectious agents. Validation of the FilmArray technology was achieved through development of a panel of assays capable of identifying 21 common viral and bacterial respiratory pathogens. Initial testing of the system using both cultured organisms and clinical nasal aspirates obtained from children demonstrated an analytical and clinical sensitivity and specificity comparable to existing diagnostic platforms. We demonstrate that automated identification of pathogens from their corresponding target amplicon(s) can be accomplished by analysis of the DNA melting curve of the amplicon.

## Introduction

The ability to rapidly detect and distinguish multiple infectious organisms is critical for the accurate diagnosis of seasonal and sporadic outbreaks, emerging pathogens and agents of bioterrorism [1], [2], [3], [4]. Accurate pathogen identification allows clinicians to determine the need for additional ancillary diagnostic testing, antibacterial or antiviral therapy and can inform decisions regarding hospitalization and infection control measures [5], [6], [7], [8], [9].

Standard microbiological testing can require several days for initial identification of a pathogenic organism, and many organisms cannot be recovered using conventional techniques [10], [11]. Molecular methods, particularly the polymerase chain reaction (PCR) have expanded the range of pathogens that can be identified in clinical laboratories. However, existing diagnostic assays and technologies are either limited in scope or highly complex [12].

Although it has many advantages, the introduction of PCR into the standard clinical microbiology and virology laboratory has been associated with practical challenges [13] that have limited routine use to large hospital or reference laboratories. Specialized training and facilities are required for technicians to perform PCR-based testing. For example, physically separated locations for sample preparation, formulation of reagents, reaction set up and amplification are needed to minimize the potential for contamination which can lead to false positive results. Even simple PCR platforms have instrument requirements that may challenge the capacity of clinical laboratories [14], [15], [16], [17].

PCR assays for infectious disease range from the relatively simple, in which a pathogen is identified by the detection of a single positive amplification product, to multiplex assays for groups of pathogens [18]. Multiplex PCR allows the potential amplification of many nucleic acid targets within a single reaction [19], [20], [21], [22], [23], [24], [25], [26], [27], [28], and in theory is an ideal method for multi-pathogen detection. However, multiplex PCR has several practical limitations. Non-specific products generated through primer-primer interactions interfere with amplification of the actual targets and decrease sensitivity. In addition, as more true targets are amplified, it becomes increasingly difficult to distinguish the amplification products, although both bead-array and microchips have been used to accomplish this [29], [30], [31], [32], [33]. These constraints have limited the ability of multiplex PCR to interrogate a large number of targets, and reports of more than 8-deep multiplexing are uncommon [34], [35], [36], [37], [38], [39], [40].

Despite the limitations, multiplex PCR strategies have already demonstrated clinical utility, particularly for detection and identification of pathogens causing respiratory tract infection [17], [35], [38], [39], [41], [42]. Multiplex PCR is an attractive diagnostic option for respiratory infections for several reasons. The differential diagnosis of respiratory infections, such as bronchiolitis and pneumonia, includes a large number of potential pathogens that cause similar signs and symptoms [43], [44], [45]. Further, conventional diagnostic testing for respiratory pathogens is limited by poor sensitivity or prolonged turnaround time of antigen or culture-based testing. Finally the emergence of novel pathogens that may result in severe disease, such as SARS and swine-origin influenza A (H1N1-2009), requires the availability of diagnostic testing with enough flexibility to introduce new targets rapidly.

Sensitivity of detection is another important consideration when developing diagnostic tests, as even very low levels of pathogen can cause disease. Nested PCR is an exquisitely sensitive methodology in which a target is amplified in a two-step process. In the first stage, a template is amplified using a pair of “outer” primers. This PCR product is diluted and subjected to a second stage amplification using primers located within the first PCR amplicon. The second stage product can be detected by real-time or end-product analysis. Nested PCR increases sensitivity over conventional PCR due to the ability to perform up to 50 or 60 total cycles of PCR. Specificity of nested PCR is similar to that of probe-based assays, as all 4 primers must match the template [1], [46], [47], [48], [49], [50], [51]. Although the use of nested primer sets was described very early in the history of PCR [52] it has not been widely deployed in clinical settings because, in most systems, it is an open-tube procedure that is highly subject to self-contamination.

An integrated system that can interrogate a clinical sample for a broad range of pathogens is highly desirable in both diagnostic laboratory and clinical settings [12], [17], [53]. Here we describe a novel diagnostic platform, the “FilmArray®”, which combines automated sample preparation, nucleic acid extraction and PCR-based detection of 31 separate targets from a single unprocessed sample in one hour. It combines nesting and multiplexing of the PCR (referred to here as nested multiplex or “nmPCR”) together with DNA melting curve analysis [54] to detect and distinguish multiple pathogens simultaneously. Because the sample manipulations and reactions are performed in an enclosed pouch, there is low risk of laboratory contamination. We detail our validation of the system using cultured respiratory pathogens and demonstrate its utility using clinical samples obtained from children with respiratory infections. The FilmArray and the FilmArray Respiratory Panel (RP) pouch have since received FDA clearance for use as an in vitro diagnostic (IVD) device.

## Methods

### The pouch

**Figure 1:**
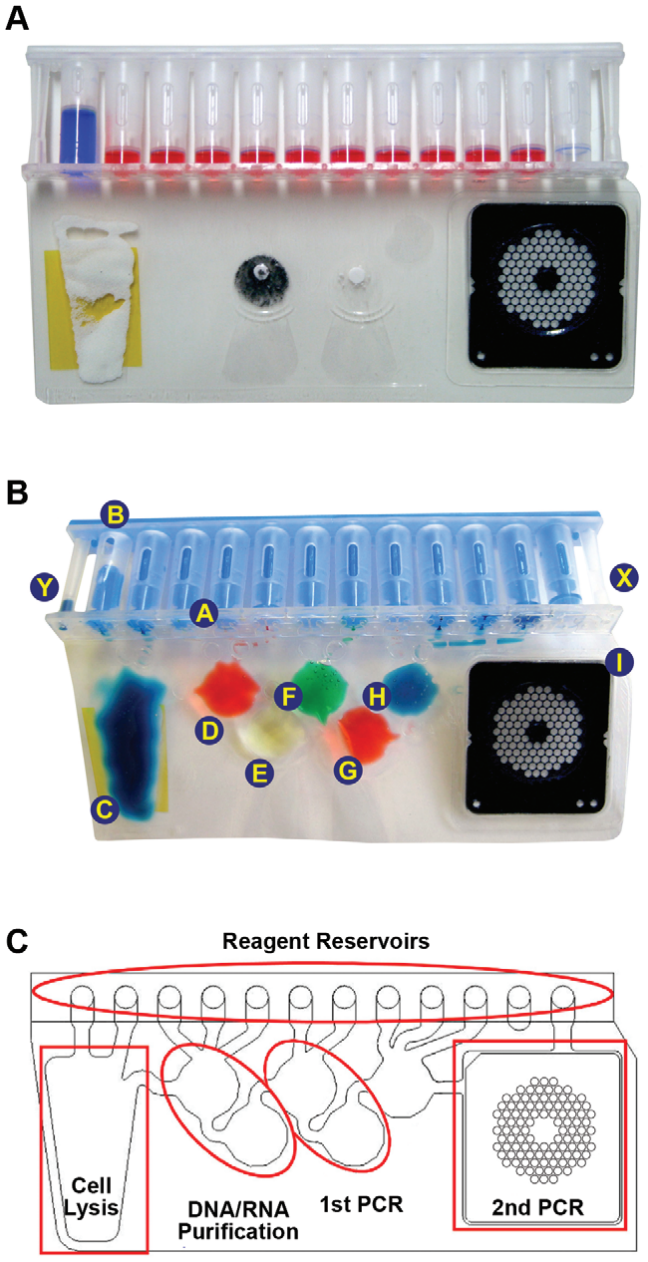
FilmArray pouch. (***A***) A FilmArray pouch was injected with mock sample (here colored blue for illustrative purposes) in the left side injection port and hydration solution (colored red) in the right side injection port. (**B**) The blisters of a FilmArray pouch were filled with different coloring (and the channels between the blisters heat sealed shut). In this pouch the plunger tree was made from plastic dyed blue. The fitment and film are normally at right angles to each other; for clarity the pouch has been flattened. (**C**) A schematic of the pouch showing a trace of the blisters, channels and array wells (black) and the functional areas of the pouch (red).

Each FilmArray pouch is comprised of an injection molded polypropylene reservoir (the “fitment”, 120 mm long, 10 mm wide, 25 mm high, “A” in Figure 1B) heat welded to two sheets of a polyester/polypropylene film containing a copolymer adhesive layer. The sheets of film are welded together using heated plates to form the pattern of channels and “blisters” (“C” through “H” in Figure 1B) comprising the sample processing stations and an area containing a 102-well array for the second stage PCR. The fitment contains 12 reservoirs (6 mm inner diameter on 9 mm spacing) that hold the biochemical reagents. During pouch manufacture three additional reagents are inserted into the appropriate blisters of the pouch and the film is sealed shut under vacuum. Ceramic beads are inserted into the sample lysis blister (“C” in Figure 1). A lyophilized pellet of silica-magnetic beads is inserted into blister “E” (Figure 1). A lyophilized pellet of the oligonucleotides (Idaho Technology, Inc. (ITI)) used in the first stage multiplex PCR is inserted into blister “G”.

**Figure 2:**
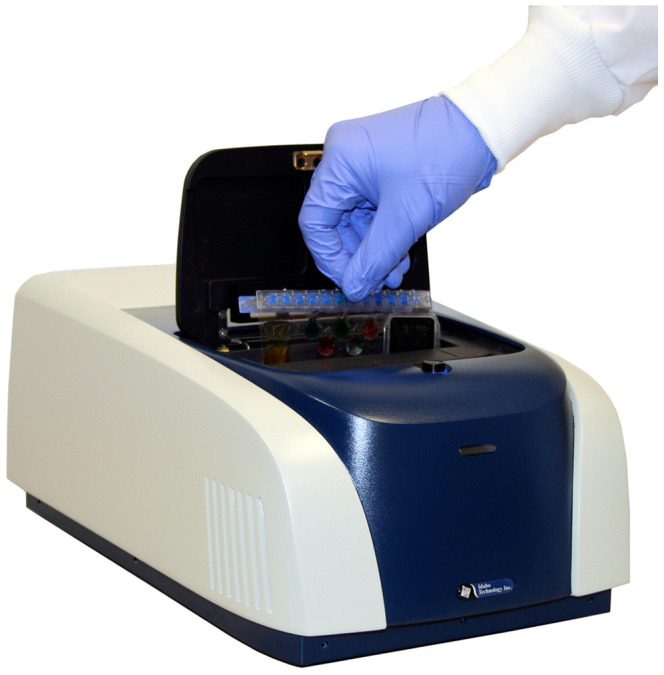
Schematic of second stage PCR mix entering the array. The layers of film and adhesive attaching the array to the pouch are separated to show the flow of liquid into the cells of the array (figure is not to scale). From the top the layers are: 2^nd^ pouch film, 1st pouch film, array adhesive layer (orange), pricked cover film, array (black, with wells), and array cover film. All of the actual layers are transparent except for the array itself. Second stage PCR primers are spotted into the cells during manufacture and air-dried (Methods). Arrows show the flow of PCR master mix (without primers) entering the array through a hole cut in the 1^st^ pouch film.

The second stage PCR array is manufactured from 0.5 mm thick black polycarbonate plastic. 102 wells of 1 µl volume each are drilled into the array (“I” of Figure 1B and Figure 2). Laminating film is heat-sealed to the back of each array and 96 arrays are placed on a platen on the bed of a piezo-electric microarraying instrument (Nano-Plotter NP2.1e, GeSiM, Großerkmannsdorf, Germany). The second stage primer sets are dispensed into the wells of the array using the standard GeSiM Nano-Tip. After spotting, the arrays are sealed with a second layer of laminating film containing a matching array of holes (Figure 2) and then attached to the outside of the pouch (“I” in Figure 1) using pressure sensitive adhesive film.

All of the other biochemical reagents are freeze-dried into the 12 wells of the fitment. Moving from left to right in Figure 1 the wells contain:

Well 1: Process control material (*Schizosaccharomyces pombe* cells).

Wells 2, 3, 4, 5: Wash buffer

Well 6: Nucleic acid elution buffer.

Well 7: Reverse transcription/first stage PCR master mix:

Well 8: Dilution buffer

Wells 9, 10: Second stage PCR master mix: containing LCGreen® Plus+ (ITI),

Well 11: empty

Well 12: Overflow reservoir for the second stage PCR mix.

After these reagents are loaded into pouches a “plunger tree” (“B” in Figure 1B) is inserted into the fitment and the pouches are placed in a Genesis Lyophilizer (VirTis, Gardiner, NY). At the end of the lyophilization cycle, while the pouches are still under vacuum, the plunger tree is pushed down into the fitment so as to preserve the vacuum in each of the wells. To maintain the vacuum (in the fitment and blisters) during long term storage, pouches are packaged in a cylindrical aluminum can and sealed under vacuum inside an aluminized polyester bag. Vacuum storage of the pouch serves three functions. It helps to maintain the integrity of the freeze dried reagents during long term storage, it enables sample and hydration solution to be delivered in an unmetered fashion and it minimizes the formation of air bubbles in the pouch, which can be difficult to control or remove from microfluidic systems [55].

### The instrument

**Figure 3:**
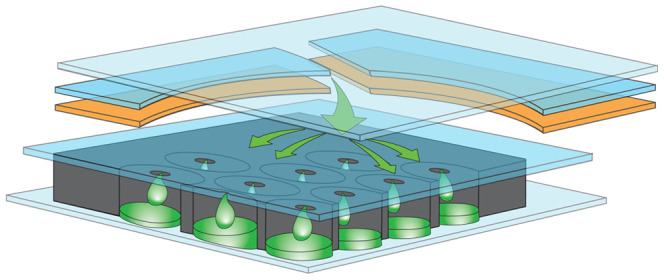
FilmArray instrument with pouch being loaded.

The FilmArray instrument is 39.1 cm long×25.4 cm wide×16.3 cm high, weighs 8.2 kg (Figure 3) and runs on 120–220 V AC power. It communicates with PC side software through USB and IEEE 1394 (Firewire™) cables. The instrument contains two Peltier devices to thermocycle the first and second stage PCR reactions, a blue LED to illuminate the second stage PCR array and a digital camera to record fluorescence generated in the second stage PCR.

The movement of liquid through the pouch is controlled by three pneumatic elements within the instrument. Pistons (located behind “B” in Figure 1) depress the plungers in the top of the fitment and thus inject reagents from the fitment into the pouch. Silicone bladders inflate over the pouch blisters to move liquid between blisters. Blunt edged pistons (“Hard seals”), positioned over the channels connecting the blisters control liquid movement between the blisters. Electronically controlled valves activate the pistons, bladders and hard seals in a coordinated pattern to regulate the flow of liquid through the pouch.

### Nested PCR assay design

Organism related sequence information (complete genomes, gene sequences and partial gene sequences) was obtained from NCBI (http://www.ncbi.nlm.nih.gov). Regions containing large conserved segments of protein sequence or conserved 5′ UTR sequences (for human Rhinovirus (HRV) and *Bordetella pertussis* (*B. per*)) were selected for assay targets (Table 1). Nearest neighbor information was obtained through the NCBI Taxonomy database http://www.ncbi.nlm.nih.gov/Taxonomy [56], [57] and used in alignments to ensure specificity of assay design. Assays were designed with a first stage PCR amplicon length of 145 to 450 (median 193) base pairs (bp). Primer sites for the second stage PCR amplicons were located within the first stage PCR amplicon and were designed to generate an amplicon of approximately 49 to 180 (median 88) bp. Primer lengths were between 17 and 37 bp and the annealing temperatures ranged from 54°C to 72°C. Degeneracy of up to 64-fold was used in some primer designs in order to accommodate sequence diversity but degenerate bases were not allowed within 4 bases of the 3′ end. In order to avoid higher order degenerate primers, some assays utilize multiple independent primers. When multiple primers were needed to achieve full coverage of diverse organisms, they were either separated as pairs in distinct wells of the array, or combined as a multiplex in single wells.

**Table 1:** FilmArray RP Pouch Pathogens, Gene Targets and LOD_95_.

Organism	Gene Target(s)	Strain^a^	LOD_95_^b^
AV	Hexon	Type 1	300
BoV	NP-1	Clinical Sample	4000
*B. per*	Toxin	A639	4,000
*C. pne*	ompA	TW183	3000
CoV 229E	Polymerase	VR-740	4
CoV HKU1	Nucleoprotein	PCMC 6123	1.9×10^6^
CoV OC43	Nucleoprotein	VR-759	600
CoV NL63	Nucleoprotein	NR-470	5
EV	5′ UTR	Echovirus 6	30,000^c^
hMPV	Nucleoprotein	hMPV-16/IA10-2003 Type A1	2
HRV	5′UTR	1A	1
Flu A (H1N1)	Matrix^d^, NS1^d^, HA1	A/Brisbane/59/07	200
Flu A (H1N1- 2009)	Matrix^d^, NS1^d^, HA1-2009	A/SwineNY/03/2009	100
Flu A (H3N2)	Matrix^d^, NS1^d^, HA3	A/Wisconsin/67/2005	5
Flu B	Hemagglutinin	B/FL/04/06	60
*M. pne*	Toxin	M129 – Type 1	30
PIV 1	Hemagglutinin	Type 1	500
PIV 2	Fusion	Type 2	10
PIV 3	Fusion	Type 3	10
PIV 4	Fusion	Type 4a	5,000
RSV	Matrix	RSV Type A	2

a See Table S1 for the source of the organisms.

b LoD concentrations are expressed in CFU/ml and TCID_50_/mL for bacteria and viruses respectively except for *C. pne* and BoV (DNA copies/mL) and CoV-HKU1 (RNA copies/ml) respectively (Methods).

c The LoD for Enterovirus (30,000 TCID_50_/ml) is based on positive results for the Entero1 or Entero2 assays. A final result of Human Rhinovirus/Enterovirus based on the combination of 6 different assays (HRV1–4, Entero1 and Entero2) can be obtained at much lower concentrations (∼300 TCID_50_/mL).

d The Flu A Matrix and NS1 gene assays are referred to as “pan1” and “pan2” respectively in the text.

AV, Adenovirus; B. per, Bordetella pertussis; BoV, Bocavirus; *C. pne*, *Chlamydophila pneumoniae*; CoV, Coronavirus; EV, Enterovirus; FluA, Influenza A ; FluB, Influenza B; hMPV, Human metapneumovirus ; HRV, human Rhinovirus; *M. pne*, *Mycoplasma pneumoniae*; PIV1–4, Parainfluenza viruses 1–4; RSV, Respiratory Syncytial Virus.

1^st^ and 2^nd^ stage assays were initially tested separately to ensure that they produced the expected amplification product, and that RNA assays were dependent on the presence of reverse-transcriptase in the reaction mix. An additional criterion was that all 1^st^ and 2^nd^ stage primers must function well at the same annealing temperature. First and 2^nd^ stage assays were then combined to form a singleplex, nested assay and tested for efficiency, sensitivity and specificity using quantification cycles (C_q_, [58]) as the readout. The first stage primers from nested assays with good overall amplification efficiency were subsequently combined as a multiplex. The C_q_s for each assay performed either as a singleplex or multiplex in the first stage were then compared. For most assays the C_q_s were quite similar. For the few cases where this was not true, moving one or the other primer a few nucleotides along the target sequence was sufficient to rescue the performance in the first stage multiplex PCR. After each redesign all assays were retested as described.

### Performing a FilmArray run

#### Pouch preparation

The freeze-dried reagents in the fitment are resuspended with hydration solution using a 3 ml syringe fitted with a blunt metal cannula. The cannula is inserted into the hydration port (“X” in Figure 1B) where it breaks a septum in the port. The vacuum in the fitment draws liquid to fill wells 2 through 11 (∼80 µl each). Sample to be tested (a nasopharyngeal aspirate (NPA) in PBS or a nasopharyngeal swab (NPS) in viral transport medium) is mixed with two volumes of a denaturing sample buffer and injected into the pouch through the sample injection port (“Y” in Figure 1B). Well 1 draws in 300 µl of this mixture). The loaded pouch is then inserted into the FilmArray instrument, and the pouch and sample are identified to the instrument by the operator using a hand-held bar code reader. After the run is started all further steps are performed by the instrument.

Sample injection into the pouch is performed in a biosafety cabinet following the appropriate biohazard guidelines for working with potentially infectious samples. For the RP pouch, the FilmArray instrument may be operated on a laboratory bench or inside a biosafety hood.

#### Sample lysis

Well 1 of the fitment contains the sample together with *S. pombe* yeast cells freeze-dried into the well as a processing control. A piston adjacent to the fitment forces the contents of well 1 into the large cell lysis blister (“C” of Figure 1) where viruses and bacteria (as well as the yeast control material freeze dried into well 1) are mechanically disrupted by 60 seconds of vigorous agitation with ceramic beads. The force for this disruption comes from a rotating metal bar located behind the plane of the pouch shown in Figure 1.

#### Nucleic acid purification

Total nucleic acid in the sample is isolated by moving the sample lysate across the silica-magnetic beads particles present in well “E”. A retractable permanent magnet (located behind blister “E” in Figure 1) is used to collect the magnetic beads in this blister. Magnetic beads are concentrated in blister “E” with the magnet and washed 3 times with buffer from wells 3, 4 and 5. After the nucleic acid binding step, blister “C” serves as a waste container for later steps in the process. Nucleic acid is eluted with buffer brought in from well 6 and all of the eluted material is moved through blister “F” into blister “G” where the mixture serves to resuspend the freeze dried pellet of first stage PCR primers.

#### cDNA synthesis and outer multiplex PCR

Reverse transcription and first stage PCR occur in blisters “F” and “G”. PCR master mix (containing the reverse transcriptase) from well 7 of the fitment is pushed into blister “F”. Bladders push both blisters against a Peltier device behind the pouch. A mechanical hot start is achieved by holding the contents of the two blisters separate (using a hard seal between them) until they reach 54°C. Reverse transcription occurs during an initial 3 minute hold at 54°C. The first stage PCR consists of 26 cycles of 94°C for 4 seconds followed by 60°C for 19 seconds. During reverse transcription and PCR cycling the contents of the reaction are mixed by moving the liquid between blisters “F” and “G”. At the end of cycling the reaction is diluted approximately 225-fold into second stage PCR master mix by two successive dilution steps, first with TE buffer from well 8 and then with PCR master mix from wells 9 and 10.

#### Nested PCR

The second stage PCR occurs in the wells of the array (“I” in Figure 1). A mechanical hot start is achieved by holding the second stage PCR master mix/diluted template mixture at 90°C while the array is heated to 75°C. The array is then flooded with this mixture (shown schematically in Figure 2) which hydrates the inner primers in their individual wells. To seal the PCR wells shut, a clear plastic bladder in the instrument is inflated over the array after it is flooded. Second stage PCR cycling conditions are 94°C for 4 seconds and 63°C for 19 seconds for 30 cycles. Ramp rates are approximately 1.7°C/sec. Images can be acquired once per PCR cycle in order to generate conventional real-time PCR amplification curves.

#### Amplicon melt analysis

After the final PCR cycle the sample is held at 63°C for 5 sec followed by a linear ramp in temperature from 68°C to 95°C at a nominal rate of 0.5°C/second. Images are acquired 10 times per second.

### FilmArray data analysis

The FilmArray instrument is capable of collecting fluorescence images and corresponding temperature data during the temperature ramp performed after the second stage PCR. The melt curve, defined as the average fluorescence intensity of each well as a function of temperature, is the basis for the automated organism detection algorithm described below. During the development of the system, the instrument was also programmed to acquire images once per PCR cycle in order to generate conventional real-time PCR amplification curves and corresponding C_q_ values. However the amplification data are not used in the automated organism calls for the commercial FilmArray system (see Results).

For the automated analysis, a hierarchy of calls is made: first for individual wells, then for individual assays (when specific primers are replicated in multiple wells of the array) and finally for each organism. This analysis is first performed on the control assays. If the controls return positive results, the analysis proceeds to the pathogen assays and the results are reported. If controls assays return a negative result, the run is declared ‘Invalid’ and no organism results are reported.

For each well, curve shape and peak location analyses of the melt curve are used to make a “Positive” (amplicon present) or “Negative” call. If two or more replicate wells are Negative for any one assay, then assay is called “Negative”. Next, if two of the melting temperatures (T_m_s) for positive replicates are within assay-specific limits (see Results) then the software assigns a positive call to the assay. For organisms with a single associated assay the final test result of ‘Detected’ or ‘Not Detected’ is based on the assay call. For influenza A and Rhinovirus/Enterovirus, the final test result is based on the integration of all associated assays.

### Sources of viruses, bacteria and clinical samples

Viruses and bacteria used in this study are indicated in Table S1. Growth, quantification and verification of viral and bacterial cultures were performed by Zeptometrix (Buffalo, NY). Bocavirus (BoV) and Coronavirus (CoV) HKU1 could not be grown in culture. Instead, well-characterized clinical specimens were utilized and quantified in copies per ml by real-time PCR against a standard curve of synthetic template.

Residual clinical NPA specimens (stored frozen at −80°C) came from children younger than 18 years who had NPA collected for respiratory viral testing by direct fluorescent antibody (DFA) and culture at Primary Children's Medical Center (PCMC), Salt Lake City, UT between 2006 and 2008. Approximately half of the NPA specimens chosen for analysis were negative by DFA and viral culture. FilmArray testing was performed at both PCMC and ITI. PCR results were not used to inform clinical management or reported to microbiology technicians performing DFA and viral culture.

FilmArray data used for tuning the melt calling algorithm were acquired at sites performing beta testing of the instrument. The data used to validate the algorithm were acquired during clinical trials of the FilmArray system and RP pouch at the Medical University of South Carolina (Frederick S. Nolte, PhD), Detroit Medical Center (Hossein Salimnia, PhD), and Children's Medical Center of Dallas (Beverly Rogers, M.D.).

The institutional review boards of the University of Utah and PCMC approved this study and granted a waiver of informed consent because the patient samples were de-identified. All external clinical studies were performed with appropriate IRB approval. Data from these sites were de-identified before being sent to Idaho Technology.

### Direct Fluorescent Antibody testing and viral culture

The PCMC microbiology laboratory performs DFA for seven respiratory viruses: Influenza A (FluA), Influenza B (FluB), Respiratory Syncytial Virus (RSV), Parainfluenza viruses 1–3 (PIV 1–3) and Adenovirus (AV) using a panel of DFA assays (Simulfluor respiratory screen, Light Diagnostics, Temecula, CA) with reflex to viral culture. Human metapneumovirus (hMPV) is detected with a specific hMPV monoclonal antibody (Diagnostic Hybrids, Athens, OH).

Viral cultures are performed using a single cell line (R-Mix-Too; Diagnostic Hybrids) with an exit stain at 72 hours. The sensitivity of DFA testing, compared with viral culture, was 90% for FluA, 72% for FluB, 99% for RSV, 77% for PIVs, and 92% for hMPV, with specificity of 90% for all of the viruses in the PCMC laboratory [59], [60]. Technicians at ITI and PCMC were blinded to DFA results while evaluating NPA samples using FilmArray. Reproducibility was not evaluated in this study.

### Statistical analysis of the FilmArray versus DFA results

McNemar's test is used to compare the DFA results to the FilmArray results [61]. The test compares the number of discordant results, shown in the off diagonals of the paired 2×2 table (Table 2), to estimate the probability that the two methods have equal sensitivity and specificity. We chose to use the terms positive % agreement and negative % agreement to report the test characteristics of the FilmArray respiratory panel. Positive percent agreement (sensitivity) is the percent of time that FilmArray RP detected a virus when DFA detected it. Similarly, negative percent agreement (specificity) is the percent of time that FilmArray RP did not detect a virus when DFA did not detect it.

**Table 2:** Comparison of FilmArray RP to DFA.

	FilmArray RP	DFA		Positive Percent Agreement	Negative Percent Agreement	Discordance P Value^c^
		Pos^a^	Neg^a^	(95% CI)^b^	(95% CI)^b^	
AV	Pos	22	32	84.6 (65.1–95.6)	89.4 (85.4–92.6)	<0.001
	Neg	4	270			
hMPV	Pos	4	10	66.7 (22.3–95.7)	96.9 (94.4–98.5)	0.021
	Neg	2	312			
Flu A	Pos	14	2	100 (76.8–100)	99.4 (97.7–99.9)	0.50
	Neg	0	312			
Flu B	Pos	1	8	100 (2.5–100)	97.6 (95.2–98.9)	0.008
	Neg	0	319			
PIV1	Pos	6	0	54.5 (23.4–83.3)	100 (98.8–100)	0.063
	Neg	5	317			
PIV2	Pos	10	8	90.9 (58.7–99.8)	97.5 (95.1–98.9)	0.039
	Neg	1	309			
PIV3	Pos	19	28	95.0 (75.1–99.9)	90.9 (87.1–93.9)	<0.001
	Neg	1	280			
RSV	Pos	37	9	94.9 (82.7–99.4)	96.9 (94.2–98.6)	0.035
	Neg	2	280			

a Positive or Negative test result comparing FilmArray RP (new test) to DFA (reference standard subject to error). (N = 328)

b Clopper-Pearson 95% confidence Interval.

c McNemar test, comparing discordant cells (FilmArray positive, DFA negative) vs (FilmArray negative, DFA positive).

If the sensitivity and specificity of the two methods are equal, the two off diagonals should be approximately equal and the estimated probability of being the same should be high. If one method is more sensitive or specific than the other, one of the off diagonal cell counts would be larger than the other and the estimated probability that the two methods have the same sensitivity and specificity would be low. The test does not provide the user with the information to determine that one method is more sensitive or the other is more specific, rather only gives them the power to say that they are different.

## Results

### Optimization of the FilmArray chemistry

Initial development of the system was performed using DNA and RNA targets from *Saccharomyces cerevisiae* and *Schizosaccharomyces pombe*. To optimize the amplification of RNA targets we used intron-spanning primers to detect mRNAs and to exclude genomic DNA. Nested assays developed on conventional PCR instruments were moved into the FilmArray platform. Whole organisms or purified nucleic acid were tested in different steps of the pouch to evaluate both the nucleic acid purification and nmPCR portions of the process.

To maximize the sensitivity of the system, we determined the number of cycles in the first stage PCR that are needed to enter the plateau phase of the reaction [62], [63]. For the first 25 cycles of the 1^st^ stage multiplex reaction, amplification of specific product is efficient, and each additional 1^st^ stage cycle reduces the C_q_ in the nested reaction by one. After this point there is diminished efficiency of the first stage PCR and for this reason, the first stage PCR is run for 26 cycles.

The completed first stage PCR mixture is diluted and then mixed into fresh PCR reagents. We determined empirically that two successive dilutions of ∼15 fold were necessary and sufficient to minimize primer carryover from the first stage PCR. Dilution of less than 100 fold generated nonspecific amplification products in the second stage PCR. Dilution of more than 300 fold caused a reduction in sensitivity.

The second stage PCRs are performed in individual wells of a high-density polycarbonate array (“I” in Figure 1 and shown schematically in Figure 2). The format of the array is analogous to that of a microarray, in which analyte assays are spatially separated for identification. In contrast to a standard microarray, PCR amplification is performed in each well. Specific primers that nest within a particular first stage PCR amplicon are dried into each well; each assay is present in triplicate within the array. The double-stranded DNA binding dye LCGreen® Plus [54], [64] is used to monitor fluorescence changes during PCR amplification and during a post-PCR amplicon melt.

The FilmArray pouch incorporates three controls to assess the performance of key steps in the system. As an RNA process control, the yeast *S. pombe* is freeze dried into the first well of the fitment. Outer and inner primers targeting a spliced mRNA from *S. pombe* are included in the pouch. A positive result for this assay provides evidence that all steps of the pouch completed correctly. A double stranded DNA oligonucleotide in the first stage PCR master mix and the appropriate nested assay monitors all steps from the first stage PCR forward. A different DNA oligonucleotide is spotted into the array along with the appropriate forward and reverse primers to monitor the second stage PCR.

### Development of respiratory pathogen assays for the FilmArray

In parallel with the development of the FilmArray instrument and pouch we designed a panel of assays to detect viruses and bacteria known or suspected to cause upper respiratory tract infection in humans. Pathogens were chosen in consultation with pediatric infectious disease experts (AJB, CLB). Primers were designed to amplify conserved regions of the targets using standard software and alignment tools. The assays were initially optimized in conventional PCR instruments, using as template either organism from reference collections or pediatric NPA samples that tested positive for respiratory viruses by conventional (DFA) testing or the assays described here. Sequencing was used to confirm target identity. A successful set of assays for 23 targets from 21 pathogens was transferred to the pouch. The final FilmArray RP pouch contains 61 primers in the outer multiplex (of which four are for controls) and 31 inner second stage PCR assays (spotted in triplicate on the array with nine empty wells as negative controls). Pathogens and gene targets in the optimized RP pouch are listed in Table 1. The yeast RNA process control and the second stage PCR control are used to catch failures in the different steps of the pouch.

### Detection of virus and bacteria using the FilmArray instrument and pouch

**Figure 4:**
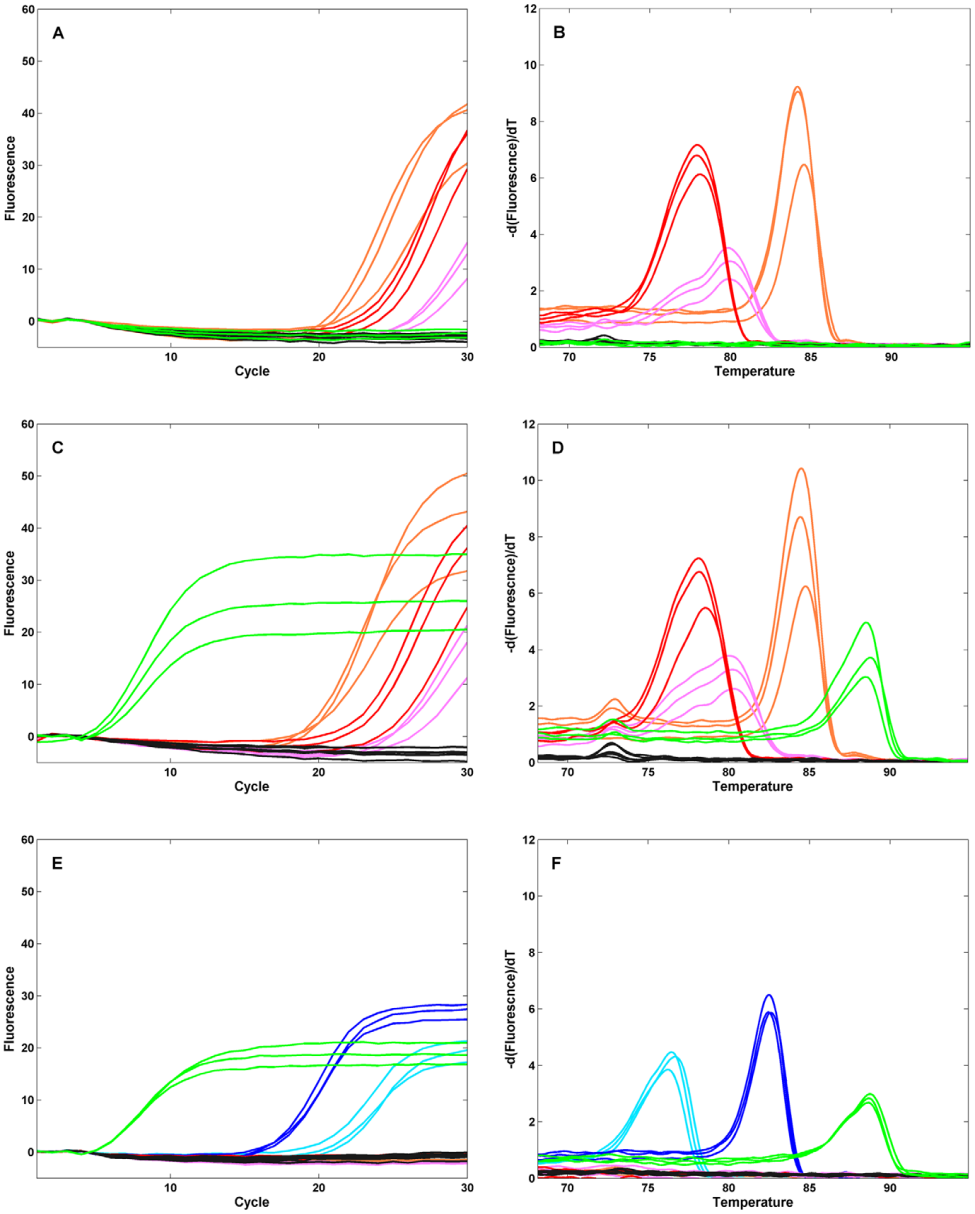
Real-time amplification and melt curves from the array. Respiratory Pathogen pouches were injected with viral transport medium spiked with 200 TCID_50_ FluA H1-seasonal (panels **A** and **B**), 4×10^6^ cfu B. per and 200 TCID_50_ FluA-H1 (panels **C** and **D**), or 4×10^6^ cfu B. per (panels **E** and **F**) and run on the FilmArray instrument. Real time amplification curves (panels **A** and **C** and **E**) and post-amplification melt curves (panels **B** and **D** and **F**) for selected wells on the array are shown. Assays are spotted in triplicate: FluA-pan1 (orange), FluA-pan2 (pink), FluA-H1-pan (red), FluA-H3 (black), B. per (Green), Yeast RNA process control (dark blue), Second stage PCR control (light blue). For clarity the controls are shown in panels **E** and **F** only.

Typical amplification and melt curves generated using a research version of the FilmArray instrument and RP pouch are shown in Figure 4. FluA H1 virus (200 Tissue Culture Infectious Doses-50% (TCID_50_)) injected into an RP pouch produced amplification products in the wells of the array containing PCR primers specific for all of the FluA H1 specific targets (Figure 4A). Melting curves generated from wells for a given assay have T_m_s that are characteristic of the amplicons from those targets (Figure 4B). The FluA H3 assay, which should not be positive for this virus, does not show evidence of amplification or melt peaks. Figure 4E and F show the result of injecting a very high level of another organism, *B. per*, into a FilmArray pouch. The amplification curves for the *B. per* assay replicates have a C_q_ of 5. This is the earliest C_q_ observed in the system and represents dilution and second stage amplification of an outer amplicon that has fully entered plateau in the first stage reaction.

FilmArray can detect multiple targets in a single assay, and in particular, detect a low-copy target in the presence of a different, high-copy target. Figure 4C and D shows the results from a pouch in which both the high-titer *B. per* and low-titer FluA were injected. The *B. per* is detected early (low C_q_), as expected. In addition, all of the Flu A target amplicons are detected, with similar C_q_s and T_m_s as found for the FluA sample alone.

Figure 4E and 4F also show the RNA process control and the second stage PCR control. The assays amplify and produce the expected characteristic melts indicating that 1) all of the different steps of the pouch performed as expected and 2) the sample did not inhibit the pouch chemistry.

### Pre-clinical evaluation of the FilmArray with pediatric NPA samples

To determine whether the FilmArray system would detect organisms in patient samples, we performed a study using pediatric NPA samples previously tested for respiratory infection at PCMC by DFA. Pre-clinical testing was performed at ITI and also during a 2-month placement of an instrument within the PCMC microbiology laboratory. Positive organism calls were made by expert users examining the amplification and melt curves. Three hundred and twenty eight samples were tested by both DFA and FilmArray. The results were compared for those viruses identified by both testing methods. When analyzed separately, similar results were obtained from both the research and clinical laboratories (data not shown) and thus combined data is presented.

**Figure 5:**
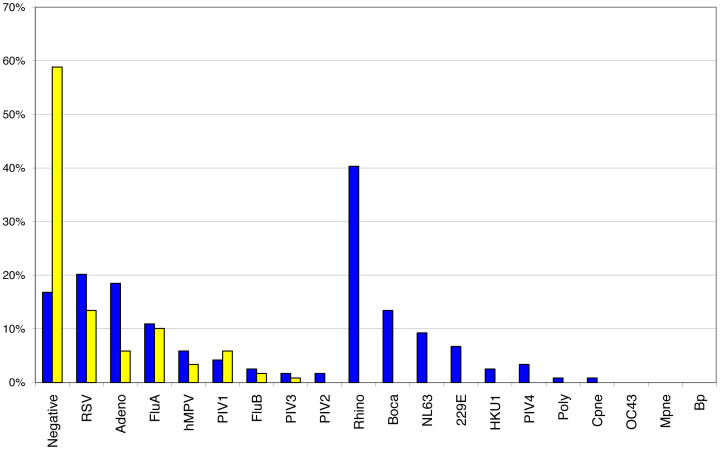
Detection rates of the FilmArray RP pouch compared to DFA. Pediatric NPA samples (N = 328) were tested either by DFA at PCMC (yellow bars) or on the FilmArray (Blue bars). The percent of samples in which no virus (Negative) or one of the indicated viruses was detected is shown. The viruses are grouped into those in which both DFA and FilmArray assays are available or only the FilmArray assay is available.

The FilmArray, with 21 respiratory pathogen assays, identified significantly more pathogens than DFA in these pediatric samples (Figure 5). FilmArray testing decreased the number of clinical samples with no pathogen identified from 63% by DFA to 19% by FilmArray (p value<0.0001). For the pathogens tested by DFA, the concordance between FilmArray and DFA testing was high. Positive percent agreement with DFA ranged from 55%–100%, although for most the agreement was >90% (Table 2). The two pathogens with the lowest percent agreement were PIV1 (55%) and hMPV (67%). Some samples that were PIV1 positive by DFA were PIV3 positive by FilmArray and were confirmed to be PIV3 by sequence analysis. Because the FilmArray testing was done retrospectively, DFA could not be repeated. For hMPV, there were too few positive samples to fully interpret discordant results.

### Using amplicon melting to automate analysis of the assay results

**Figure 6:**
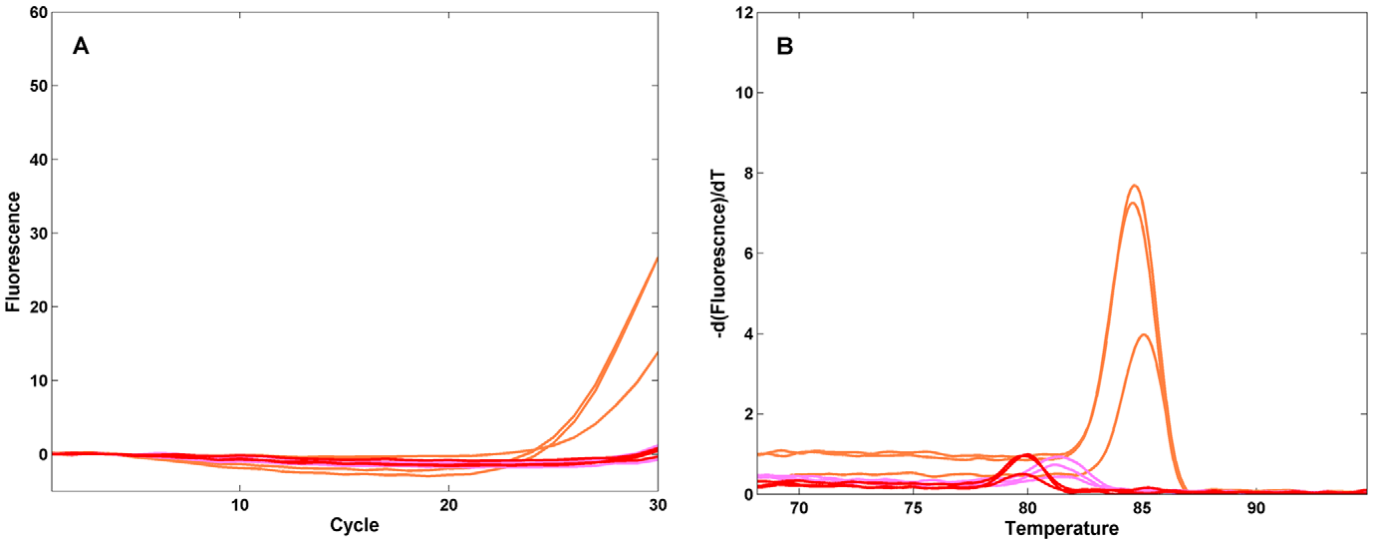
Amplification and melt curves at low target levels. Respiratory Pathogen pouches were injected with viral transport medium spiked with 1 TCID_50_ of the FluA- H1 seasonal virus used in Figure 4, and run on the FilmArray instrument. Real-time amplification curves (**A**) and post-amplification melt curves (**B**) for selected wells on the array are shown. Assays are spotted in triplicate: FluA-pan1 (orange), FluA-pan2 (pink), FluA-H1-pan (red). The ordinate scales are the same as in Figure 4.

A diagnostic system that automates the technically demanding steps of nucleic acid isolation and PCR amplification would benefit from automated analysis of the PCR results. FilmArray runs generate large amounts of data in the form of real time amplification curves and the associated melt curves. In similar systems the properties of the amplification curve are used to make a positive or negative call for that assay [65]. In the course of analyzing the FilmArray data from reference strains and clinical samples, we observed that the amplicon melt curve shapes and T_m_s were highly specific to the organism targeted by the nested PCR and thus could provide an additional filter for detecting each organism. For high or moderate titer organisms, the FilmArray system produces both robust amplification and melt curves with a high signal-to-noise ratio (Figure 4A and B). For very low titer organisms, the amplification curve is often obscured by the noise inherent in thermocycling. For example, Figure 6 shows the amplification and melt curve data from a pouch injected with a very low level FluA sample (1/200^th^ of that in Figure 4). Of the three assays in the pouch that can detect this organism, neither the FluA pan2 nor the FluA H1 pan amplification curves show a rise above baseline; only the FluA pan1 assay produced significant amplification. By contrast, a robust signal can be detected in the melt curves for the same sample (Figure 6B). The automated analysis of the melt curves produced a positive result for the FluA pan1 and pan2 assays (2 or more of the 3 replicates were positive) and a negative result for the FluA H1 pan assay (only one melt curve was positive).

To compare the sensitivity of a detection algorithm based on melt curve analysis to one based on amplification curves, we had expert users annotate a total of 18,156 amplification and melting curves as positive or negative. Automated analysis of the data using both the amplification and melt profiles produced a sensitivity of 94.7%, a specificity of 99.95%, and an error rate of 4.15% compared to the expert user's annotations. By comparison, analysis of the melt curves alone produced a sensitivity of 97.49%, a specificity of 99.6%, and a total error rate of 2.92% compared to the expert calls. The higher error rate of the combined analysis is explained by false negative calls for weak amplification curves. Therefore we proceeded to develop an automated analysis of the FilmArray data using only the melt curves.

**Figure 7:**
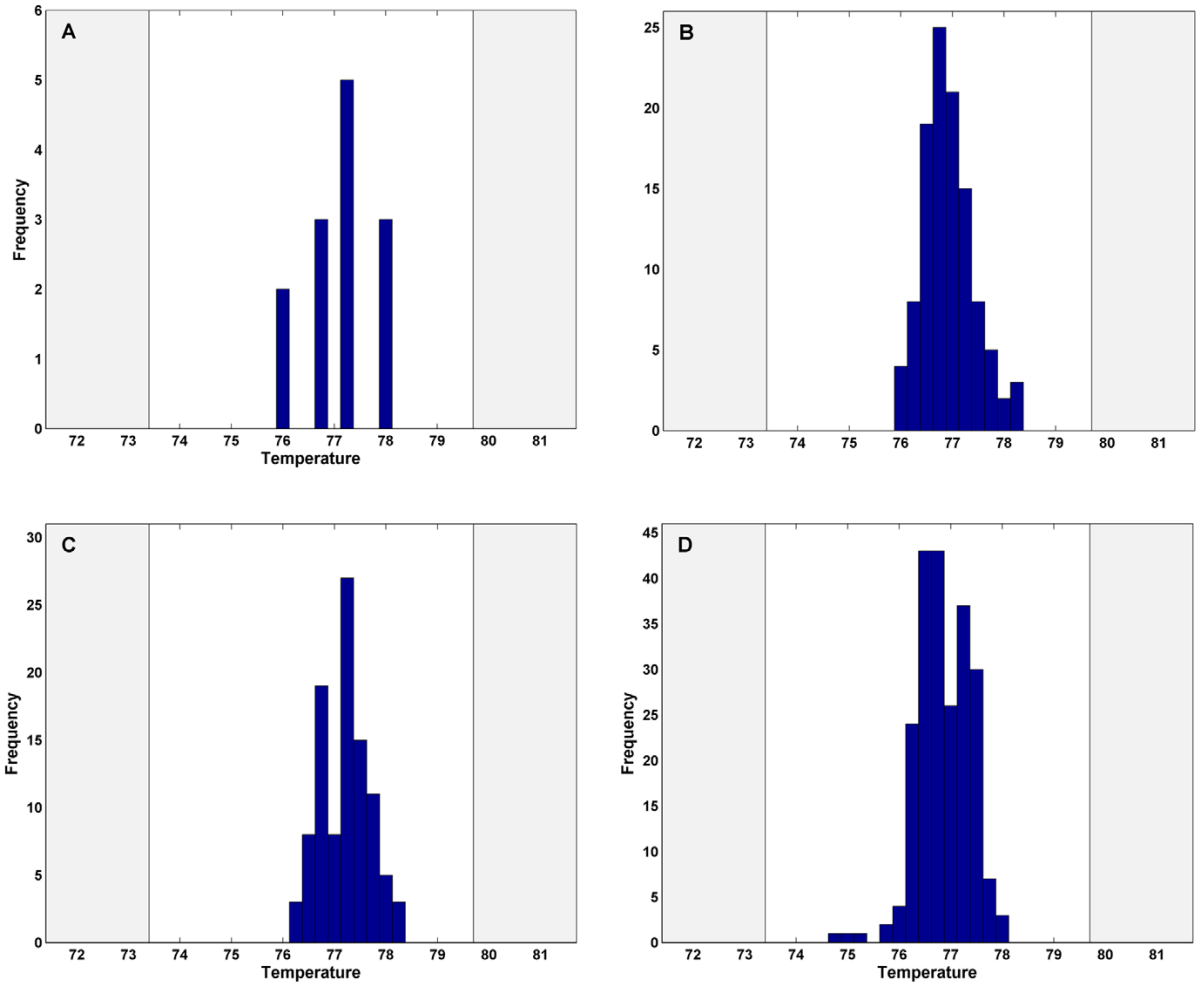
T_m_ data used to establish assay specific melt windows. Histograms of the theoretical or observed T_m_s of the hMPV assay are shown. T_m_ data for the FilmArray runs includes each of the three replicates of the second stage PCR. **A:** T_m_s calculated from 13 sequence variants published in the NCBI databases. **B:** T_m_ data generated during the system beta-testing with 37 banked hMPV-positive patient samples. **C:** T_m_ data generated during the inclusivity testing with 10 hMPV strains representing subtypes A1, A2, B1 and B2. Multiple FilmArray runs of these strains are included in this data set. **D:** T_m_ data from 74 hMPV-positive patient samples collected during the clinical evaluation.

To maximize the specificity of melt curve analysis, we determined the range of possible T_m_s for amplicons from each different organism assay. The theoretical melting temperature of a DNA sequence on the FilmArray instrument was calculated using the model (modified from [66]):
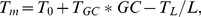
(1)where *GC* is the mole fraction of G and C bp in the sequence, *L* is the length of the amplicon, and *T_o_*, *T_GC_*, and *T_L_* are empirically fit parameters estimated using FilmArray data from samples of known sequence. GenBank was searched for sequence variants of each organism and these data were trimmed to the inner PCR product of the nmPCR. Predicted T_m_s for these variants were calculated using the mathematical model determined above (the data for hMPV are shown in Figure 7A). This distribution of T_m_s was used to establish the expected melt range for each assay. These ranges were expanded beyond the minimum and maximum predicted T_m_s to account for system variability (determined by the T_m_s of the control assays) and the T_m_s obtained from initial clinical testing (Figure 7B). To validate these predictions, the distributions of T_m_ data from testing of reference strains (Figure 7C) and from further clinical evaluations (Figure 7D) were compared to the initial melt range. The overlap in distribution of T_m_s between the different sample sets suggested that the melt ranges adequately, capture the full diversity of amplicon T_m_s for this organism. Narrowing the melt window in this way eliminates some false positives due to nonspecific amplification (data not shown).

To maximize sensitivity and specificity of the melting curve detection algorithm we optimized it using a large training dataset comprising 1566 RP pouch runs performed both at Idaho Technology (900 runs) and at external sites (666 runs) (Methods). The majority of the data generated at Idaho Technology was derived from contrived samples spiked with dilution series of the various target organisms (Table S1). Data from external sites was primarily composed of residual archived clinical samples evaluated during beta testing of the system. Using a semi-automated process, the detection algorithm was tuned so that the sensitivity and specificity are greater than 99% (Table 3, Training Set). To validate the performance of the detection algorithm, a second independent dataset consisting of 511 FilmArray runs of clinical samples was annotated by expert users. As shown in Table 3 (Validation Set), the detection algorithm is able to differentiate positive and negative melt curves with a high degree of accuracy (>99.5%).

**Table 3:** Performance of the FilmArray RP melt curve detection algorithm compared to expert interpretation.

Expert Interpretation		Melt Detector Call		Percent agreement (95% CI)
		Positive	Negative	
Training Set	Positive	37,614	231	99.39 Pos (99.31–99.47)
	Negative	141	108,529	99.87 Neg (99.85–99.89)
Validation Set	Positive	8,153	30	99.63 Pos (99.48–99.75)
	Negative	17	39,323	99.96 Neg (99.93–99.97)

Using the same training and validation data set, the FilmArray automated analysis was compared to expert calls for the assay results (Table 4). Data from the triplicate wells of each assay were combined to produce a “Positive” or “Negative” assay call. In this case the results of the automated analysis of the FilmArray RP system resulted in sensitivity and specificity >99.6%.

**Table 4:** Performance of the FilmArray RP system automated analysis as compared to expert interpretation.

Expert Interpretation		RP system Assay Call		Percent agreement (95% CI)
		Positive	Negative	
Training Set	Positive	12,596	34	99.73 Pos (99.62–99.81)
	Negative	4	35,912	99.99 Neg (99.97–100.00)
Validation Set	Positive	2,713	8	99.71 Pos (99.42–99.87)
	Negative	1	13,119	99.99 Neg (99.96–100.00)

### Determination of the analytical Limit of Detection for the FilmArray RP system

To determine at what level the PCR assays in the FilmArray system could correctly and consistently identify organisms, titered viral and bacterial respiratory pathogens were spiked into negative NPS sample matrix collected from healthy individuals or into a simulated NPS matrix consisting of viral transport medium (VTM) and a human epithelial cell line. Serial dilutions of the viruses and bacteria were spiked into NPS samples, both singly and in combinations of up to 5 organisms per sample. The spiked NPS samples were then tested on the FilmArray instrument. Quantification by TCID_50_ is a measure based on infectivity or cytotoxicity rather than number of organisms or copies of nucleic acid. LoD determined in TCID_50_/mL may not be an accurate indicator of the relative sensitivity of detection between different organisms.

An initial estimate of the system Limit of Detection (LoD_95_, or the concentration of organism that can be reliably detected in 95% or more of the samples tested) was based on the serial dilutions. Additional samples were then prepared and tested at the estimated LoD concentration and 10-fold lower to confirm that the correct organism was detected in at least 95% of the samples at LoD and in less than 95% of the samples containing 10-fold less organism. A positive organism detection was determined according to the automated analysis performed by the FilmArray software. For multi-assay organism calls such as FluA subtypes, all relevant assays were required to be positive at the LoD_95_ level. Column C in Table 1 shows the FilmArray LoD_95_ for each pathogen.

The sensitivity of detection was comparable between samples containing a single organism and those containing up to five different organisms. Subsequent clinical evaluations determined that the sensitivity of each assay was appropriate for accurate detection of clinically relevant pathogen levels in NPS specimens. It is worth noting in this regard that the LoD concentration for Coronavirus HKU1 (1.9×10^6^ RNA copies/mL) is below the published viral load detected in acute Coronavairus HKU1 infection (8.5–9.6×10^6^ RNA copies/ml during the first week of the illness [67]). A full description of the clinical evaluation of the FilmArray RP pouch for testing NPS samples, performed using the automated calling algorithm described above, will be published elsewhere.

## Discussion

In the last decade advances in diagnostic testing have led to changes in clinical laboratory evaluation that have translated into improved clinical care [60], [68], [69], [70], [71], [72], [73], [74]. Despite these advances, limitations remain in the arena of rapid testing for multiple pathogens and the ability to move molecular testing into clinical laboratories, particularly those unable to perform high-complexity testing. The FilmArray system addresses these concerns in that it has the capability for high-order multiplex testing, yet is simple to use and requires minimal hands-on time. Here we have demonstrated that the system can accurately detect and identify both DNA and RNA targets from whole organisms, including those contained within clinical respiratory specimens. The FilmArray can also effectively detect multiple targets in a single sample. We have developed and tested the performance of a clinically relevant panel of respiratory pathogens, including both viruses and bacteria, and shown good performance of the system when compared to standard laboratory methods.

### FilmArray implements nmPCR in a closed “lab-in-a-pouch” format

The FilmArray is a realization of a “Lab-on-a-Chip” or μTAS system (micro total analysis system, [75], [76]). It implements the highly sensitive and specific technique of nested multiplex PCR in an enclosed disposable, the FilmArray pouch. This enables the considerable benefits of this form of PCR to be realized in settings where even moderate contamination risk of pathogen or of amplicon is unacceptable. Ultimately, this “Lab-on-a-Chip”-type format could allow complex molecular methods to be adopted in “point-of-care” settings or field situations, where the patient presents initially to the healthcare provider [17], [77], [78], [79].

Although the FilmArray pouch manipulates relatively large volumes of liquid for a μTAS system, it shares several advantages with such systems. The steps of the system are automated which reduces operator work-load and error. The process is rapid: the time lag between one step of the chemistry and the next is measured in seconds. The physical separation of reagents between the fitment and the blisters also enables a hot start for both the first and second stage PCRs (see Methods). This eliminates the expense and inefficiency associated with using chemical or biochemical means of inhibiting Taq. A physical hot start also has the additional, unique, advantage that the reverse transcriptase is prevented from interacting with primers below the desired temperature of the reaction. This minimizes the formation of primer dimers or other nonspecific products in the deep multiplex of the first stage PCR.

The FilmArray lab-in-a-pouch is also an efficient solution to the “sample to assay” problem that many microfluidics systems must solve [80], [81], [82]. Typically, for low abundance pathogens, sensitivity comes from testing a large input sample volume. However, for multiplex testing, sample will often be limiting and to keep reagents costs down, the many individual tests must be done in small volumes. The FilmArray first stage multiplex PCR uses all of the nucleic acid recovered from the input material (100 µl of patient sample). The second stage amplification then allows specific detection of the analytes in individual 1 µl PCRs without the loss of sensitivity common in small volume PCRs.

In addition to its increased sensitivity and specificity, nested PCR simplifies the development of complex multiplex PCR panels. In order to detect viruses with great sequence diversity (e.g. AV and HRV) the first stage PCR contains moderately degenerate primers, or multiple primer sets. Unlike the common observation with single-stage multiplex PCR reactions [83], [84], we observed no loss of assay sensitivity when comparing nested PCR with the full complement of primers in the first stage versus a single set of primers in the first stage (data not shown). For every primer set that did show a loss of sensitivity, a redesign of the first stage assay was enough to restore sensitivity without perturbing other assays.

Whiley et al [85] have argued that multiple independent nucleic acid tests are required to ensure adequate sensitivity for detecting organisms that have significant sequence variation. Nesting of the PCRs allows a high level of multiplex in the first stage PCR, which addresses this concern. We have tested FilmArray multiplex designs that include 40 different assays and see no loss of sensitivity compared to single assay formats (M. Rogatcheva, unpublished data). In the FilmArray RP pouch described here, there are two pan-influenza A assays (pan1 and pan2 for the MA and NS2 genes respectively). This increases the likelihood that a novel pandemic influenza will be detected by the system (as a “non-subtypeable” FluA) because there is a high probability that either one or the other pan assay will be reactive.

nmPCR is also highly resistant to target competition (Figure 4). An organism present at very high concentration (10,000× LoD_95_, calculated from the data in Table 1) does not inhibit the detection of a second organism present at low concentration (10× LoD_95_). This occurs because the first stage PCR is not the direct readout for the presence of each analyte. Instead it gives the lower concentration organism adequate amplification boost, (i.e. enrichment) for detection in the second stage PCR.

### Detecting organism based on amplicon melts

The automated analysis of FilmArray data is robust to sequence variation in the target amplicon as well as potential instrument and pouch variation. The combination of melt detection in individual wells of the array together with the redundancy provided by the well-to-well comparison of replicate melt curves results in exceptionally sensitive and specific organism detection.

We have observed that melting curve analysis is more sensitive than amplification curves analysis for the detection of input material. The amount of data collected during the gradual temperature ramp of the amplicon melt greatly exceeds that collected during the relatively dynamic temperature cycling of the second stage PCR. The resulting melt curves have a higher signal to noise ratio than that of the amplification curves. For several reasons (e.g. loss of resolution at the high end of dynamic range, lack of a standard curve on a single sample instrument) the FilmArray C_q_ is not a meaningful measure of organism load in the sample. For this reason, amplification curve data are not reported in the commercial version of the instrument.

### Clinical utility of the FilmArray system

The initial testing of the FilmArray RP pouch with clinical samples demonstrates a successful real-world application of this technology. When compared to DFA using pediatric NPA samples, the platform showed high percent agreement. The most common reason for discordance was the detection of pathogens by FilmArray in DFA-negative samples. We believe this is due to the increased sensitivity of PCR when compared to DFA. In addition, the ability to test for a much larger panel of pathogens led to a decrease in the number of negative samples when compared to conventional testing, and increased the number of instances in which more than one pathogen was detected in a sample. Other multiplex PCR-based studies have reported similar findings [86].

The FilmArray instrument and a subset of the assays in the RP pouch have recently been cleared by the FDA for IVD use and an initial comparison of the FilmArray instrument with the xTAG RVP (Luminex Corporation, Austin TX) and conventional detection methods have been reported [87]. The clinical studies performed to support the FilmArray application to the FDA used NPS as the sample matrix. Because the data presented here were generated on a development version of the instrument and pouch we do not know the true clinical sensitivity and specificity of the FilmArray system when using NPA samples.

The advent of diagnostic platforms with the capability of medium level multiplexing [38], [39], [88], [89], [90], [91] opens up the potential for development of a set of multi-pathogen panels that are focused on a specific disease indication rather than a specific organism. FilmArray assay panels under development will target organisms associated with sepsis, meningitis, diarrhea, sexually transmitted infections, or bioterrorism, as well as genes conferring antibiotic resistance.

## Supporting Information

Table S1 Source of Virus and Bacteria used in this study.
(TIF)
