# Correction: FilmArray, an Automated Nested Multiplex PCR System for Multi-Pathogen Detection: Development and Application to Respiratory Tract Infection

**DOI:** 10.1371/annotation/468cfdcd-184c-42f7-a1d0-3b72a2f6a558

**Published:** 2011-11-02

**Authors:** Mark A. Poritz, Anne J. Blaschke, Carrie L. Byington, Lindsay Allen, Kody Nilsson, David E. Jones, Stephanie A. Thatcher, Thomas Robbins, Beth Lingenfelter, Elizabeth Amiott, Amy Herbener, Judy Daly, Steven F. Dobrowolski, David H. -F. Teng, Kirk M. Ririe

Figures 2 and Figures 3 are switched. 

