# Supplementary figures and images for: FilmArray, an Automated Nested Multiplex PCR System for Multi-Pathogen Detection: Development and Application to Respiratory Tract Infection
Source: PLoS One. 2011 Oct 19;6(10):e26047. doi: 10.1371/journal.pone.0026047 (PMC3198457; doi:10.1371/journal.pone.0026047)

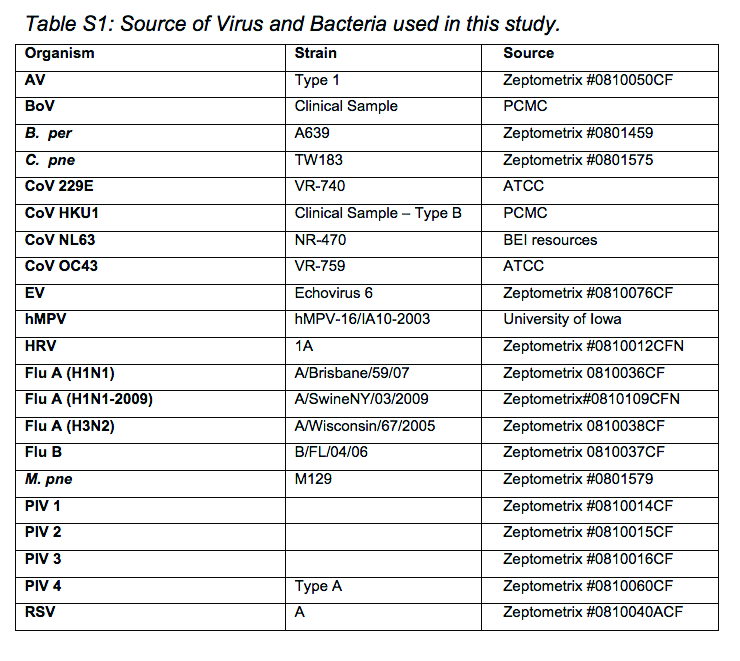

Supplement: Table S1 — Source of Virus and Bacteria used in this study. (TIF) [file pone.0026047.s001.tif]
